# Carriers of the fragile X mental retardation 1 (*FMR1*) premutation allele present with increased levels of cytokine IL-10

**DOI:** 10.1186/1742-2094-9-238

**Published:** 2012-10-13

**Authors:** Diana Marek, Stephanie Papin, Kim Ellefsen, Julien Niederhauser, Nathalie Isidor, Adriana Ransijn, Lucienne Poupon, Francois Spertini, Giuseppe Pantaleo, Sven Bergmann, Jacques S Beckmann, Sebastien Jacquemont, Goranka Tanackovic

**Affiliations:** 1 Department of Medical Genetics, University of Lausanne, Rue du Bugnon 27, Lausanne, 1005, Switzerland; 2 Swiss Institute of Bioinformatics, Quartier Sorge - Batiment Genopode, Lausanne, 1015, Switzerland; 3 Division of Immunology and Allergy, Centre Hospitalier Universitaire Vaudois, Rue du Bugnon 46, Lausanne, 1011, Switzerland; 4 Department of Clinical Neuroscience, Centre Hospitalier Universitaire Vaudois, Rue du Bugnon 46, Lausanne, 1011, Switzerland; 5 Service de Génétique Médicale, Centre Hospitalier Universitaire Vaudois, Avenue Pierre Decker 2, Lausanne, 1011, Switzerland

**Keywords:** *Fragile X mental retardation 1* (*FMR1*) gene, Fragile X-associated tremor ataxia syndrome, Immune activation, Cytokines, IL-10

## Abstract

**Background:**

Fragile X-associated tremor/ataxia syndrome (FXTAS) is an inherited late-onset neurodegenerative disorder, characterized both by neurological and cognitive deficits. It is caused by the expansion of CGG repeats (55 to 200 repeats) in the noncoding region of the fragile X mental retardation 1 (*FMR1*) gene. Abnormal immunological patterns are often associated with neurodegenerative disorders and implicated in their etiology. We therefore investigated the immune status of FXTAS patients, which had not been assessed prior to this study.

**Method:**

Peripheral blood mononuclear cells (PBMCs) were collected from 15 asymptomatic *FMR1* premutation carriers and 20 age-matched controls. Concentrations of three cytokines (IL-6, IL-8, IL-10) were measured in PBMC supernatants using ELISA assays.

**Results:**

We found a significant increase in the concentration of the major anti-inflammatory cytokine IL-10 in supernatants of PBMCs derived from premutation carriers, when compared with controls (*P* = 0.019). This increase correlated significantly with the number of CGG repeats (*P* = 0.002).

**Conclusions:**

Elevated IL-10 levels were observed in all premutation carriers, before appearance of the classical neurological symptoms; therefore, IL-10 may be one of the early biomarkers of FXTAS.

## Background

Fragile X-associated tremor/ataxia syndrome (FXTAS OMIM:300623) is a late-onset neurodegenerative disorder. FXTAS is caused by the expansion of CGG triplets (55 to 200 repeats) in the 5′-UTR of the *fragile X mental retardation 1* (*FMR1*) gene, termed premutation. The disease symptoms include: progressive intention tremor, gait ataxia associated with cognitive decline, peripheral neuropathy, and autonomic dysfunction [1]. In women, these signs are less frequent and less severe, but autoimmune dysfunction has been reported [2,3].

FXTAS is a disease with reduced penetrance: ~50% of male carriers of the *FMR1* premutation (prevalence 1 in ~500 men) and a smaller fraction of female carriers will develop FXTAS [1,4-6]. Therefore it is important to identify biomarkers that will help monitor disease progression.

At the cellular level, premutation carriers present increased amounts of expanded *FMR1* mRNA and a slight reduction in the encoded protein [7,8]. The expanded mRNA accumulates within the FXTAS-characteristic intranuclear inclusions [9], together with at least 30 different proteins including ubiquitin, stress response factors, and cytoskeletal proteins [10]. An RNA toxic gain of function was proposed as the molecular mechanism underlying FXTAS, and it was demonstrated that severity of both the clinical and the neuropathological phenotypes correlate positively both with the extent of the CGG-repeat expansion and with the increase in the amount of expanded mRNA [4]. The neuropathology of FXTAS includes significant white matter disease (spongiosis in the cerebrum and cerebellum), prominent subcortical astroglial activation, Purkinje cell loss and the presence of intranuclear inclusions in neurons and astrocytes throughout the cortex [1,4]. How this expanded RNA yields the pathological changes observed in patients remains obscure.

Evidence suggests that neuronal loss correlates with either causal or secondary excessive inflammatory response [11-14], and neuroinflammation has been implicated in a broad spectrum of neurodegenerative disorders such as Parkinson’s disease (PD), Alzheimer’s disease (AD), amyotrophic lateral sclerosis, Huntington’s disease (HD), and multiple sclerosis (reviewed in [11]). Experimental evidence shows that suppression of inflammatory processes mitigates neuronal loss in *in vivo* and *in vitro* models of neurodegenerative diseases [15-18]. Importantly, a prolonged use of nonsteroidal anti-inflammatory drugs was beneficial in both AD and PD [19-22]. Taken together, these data suggest that uncontrolled inflammation, either as an initiator or as a secondary reaction, could drive chronic and progressive neurodegenerative processes.

FXTAS is a recently described neurodegenerative disorder and almost nothing is known about the immune status of the patients. We investigated the immune profile of individuals carrying the *FMR1* premutation, having no apparent neurological symptoms. In particular, we assessed the profiles of three cytokines in their peripheral blood and compared them with those of age-matched control individuals. Our results show that increased levels of IL-10 in peripheral blood are associated with the *FMR1* premutation. This suggests IL-10 as an early characteristic of FXTAS.

## Methods

### Participants and study design

All participants signed an informed consent; the study was reviewed and accepted by the institutional review board of the University of Lausanne. Initially, we recruited 38 male individuals: 17 carriers of the *FMR1* premutation and 21 age-matched controls. The two groups of controls and carriers were defined using a cutoff value of 55 CGG repeats (Additional file 1: Table S1). Participants (*n* = 3) who experienced a recent infection (indicated by increased C-reactive protein levels) were excluded. Samples from 15 individuals adequate for the analyses were included in the carrier cohort, while the control group was composed of 20 individuals.

The following clinical phenotypes were recorded and scored in both groups (Additional file 1: Table S1): diastolic and systolic blood pressure (DBP and SBP), absence or presence of diabetes mellitus (0 and 1, respectively), hemoglobin A1c (corresponding to the percentage of glycated hemoglobin), smoking status, and treatment status defined as treatment with neuroleptic or psychotropic drugs and/or drugs intended to prevent cardiovascular diseases. The structured neurological examination was videotaped, and included the Unified Parkinson’s Disease Rating Scale (UPDRS), the International Cooperative Ataxia Rating Scale (ICARS), and the Clinical Rating Scale for Tremor (CRST). The total score of the FXTAS Rating Scale (combination of UPDRS, ICARS and CRST scales and the Singer test) [23] was used as the single clinical score.

### Isolation of peripheral blood mononuclear cells

Peripheral blood samples were collected in the presence of ethylenediamine tetraacetic acid as anticoagulant. Peripheral blood mononuclear cells (PBMCs) were rapidly isolated (within 2 hours following blood draw). The Ficoll separation procedure (Ficoll-Paque Plus; GE Healthcare Life Sciences, Uppsala, Sweden) was used to separate PBMCs and plasma, according to the manufacturer’s instructions. PBMCs were washed three times with RPMI supplemented with 10% FCS and then cultured in 12-well plates in RPMI with FCS for 24 hours. Supernatants of the cultures were collected and stored at −20°C for further analyses.

### Measurements of cytokines

Cytokines were measured first in undiluted PBMC supernatants using a Milliplex MAP kit (HCYTOMAG-60K; Merck Millipore, Billerica, MA, USA) for the detection of IL-6, IL-8 and IL-10, according to the manufacturer’s instructions. Data were acquired using a Bio-Plex 200 instrument (Bio-Rad, Hercules, CA, USA) and analyzed using Bio-Plex Manager Software (version 4.1; Bio-Rad). Since the concentration levels of IL-6 and IL-8 were at the higher end of the standard curve, we performed additional measurements to determine their levels, following the dilution of PBMC supernatants (1:150), using the ELISA kits Interleukin 6 high sensitivity human ELISA Set and Interleukin 8 high sensitivity human ELISA Set (both ImmunoTools, Friesoythe, Germany), following the manufacturer’s instructions. Measurements were performed three times for IL-6 and IL-8 and twice for IL-10.

### Statistical analyses

For each recorded characteristic, we computed the mean and standard deviation or the percentage of occurrence in both control and premutation carrier groups. We performed different statistical tests in order to assess the group effect on the set of clinical feature means or medians. For normally distributed continuous variables, such as age and FXTAS Rating Scale, we ran a one-way analysis of variance (one-way ANOVA) and retrieved the *F*-test *P* values to determine whether the phenotypes from the two groups had a common mean or a different mean at an α level of 5%. DBP and SBP measurements, as well as hemoglobin A1c concentrations, showed continuous but not normal distributions; we therefore applied a nonparametric version of one-way ANOVA, the Kruskal–Wallis test. Chi-square test *P* <5% suggests that the control group median is significantly different from the premutation carrier group. Finally, to test the group effect on smoking and treatment status, a generalized linear model for binomial distributions was applied. The outcomes were restricted to two values, which represented the occurrence or nonoccurrence of the event. The test predicts the probability of smoking or being under treatment as a function of the group (controls or premutation carriers).

Raw cytokine measurements were log-transformed (natural logarithm) to better satisfy the normality assumption underlying linear regression models and then averaged. The following analyses were all run on averaged log-transformed cytokine concentrations (stated as m-IL in figures and tables).

To assess the influence of CGG repeats on cytokine concentrations, linear regression analyses were performed modeling the cytokine levels as a function of the number of CGG repeats. A Student's *t* test and its corresponding *P* value were therefore computed to test for the significance of the β regression coefficient associated with the CGG repeats. The squared correlation coefficient *R*^2^ – which indicates the proportion of variance in one variable, explained by knowledge of the second variable – was computed. The same analysis was performed adding the age variable into the model.

Multiple regression analysis was also used to assess the relationship between the FXTAS rating scale scores and the following variables: CGG repeats and age of the patients.

To identify differences in IL-10 mean concentrations between the premutation carrier and control groups, means and standard deviations for both groups were computed and a one-way ANOVA was performed. The *P* value for the *F*-test statistic was computed.

Finally, Pearson's correlations were computed to explore whether there was a correlation between the FXTAS rating scale and the cytokine levels.

All statistical analyses were performed using Matlab® (The MathWorks Inc., Natick, MA, USA).

## Results

### Clinical assessment of the premutation carriers and controls

**Figure 1:**
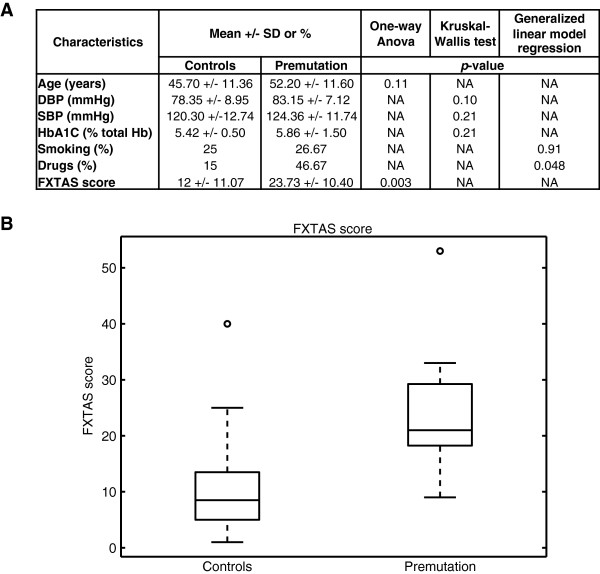
**Effect of the FXTAS premutation status on the various phenotypes studied.** (**A**) For each phenotype, the mean and standard deviation (SD) (or the percentage) were computed for the control and premutation groups. Various statistical tests were applied and their associated *P* values were computed according to the type and the distribution of the phenotypes in order to assess the effect of the clinical status. For normally distributed features (age and Fragile X-associated tremor/ataxia syndrome (FXTAS) score), a one-way analysis of variance was used. For the non-normally distributed phenotypes, such as diastolic and systolic blood pressure (DBP and SBP) and hemoglobin A1c (HbA1c) concentrations, a Kruskal–Wallis test was applied using the ranks instead of the raw values. Finally, for binary phenotypes (percentage of smokers and of individuals under treatment), a generalized linear model was applied. (**B**) Boxplot representing the distribution (median and interquartile range) of the FXTAS scores for control and premutation groups.

Several clinical and biological phenotypes were measured and compared between premutation carriers and controls (Figure 1A). The number of CGG repeats ranged from 20 to 46 in controls and from 57 to 109 in carriers. The two groups did not significantly differ in age: mean ± standard deviation = 52.20 ± 11.60 and 45.70 ± 11.36 years for the carriers and controls, respectively (*P* = 0.11). Neither did the groups differ in the presence of hypertension (DBP: 83.15 ± 7.12 vs. 78.35 ± 8.95; *P* = 0.10 and SBP: 124.36 ± 11.74 vs. 120.3 ± 12.74; *P* = 0.21), presence of diabetes mellitus (hemoglobin A1C: 5.86 ± 1.50 vs. 5.42 ± 0.50; *P* = 0.21), or smoking habits (26.67% vs. 25%; *P* = 0.91). Individuals from the carrier group were more often on medication, treated with neuroleptic or psychotropic drugs and/or drugs that prevent cardiovascular diseases (46.67% vs. 15%; *P* = 0.048), consistent with the literature [24,25].

Neurological assessment of the participants showed that the FXTAS Rating Scale scores (evaluating movement disorder symptoms) were low to normal (the highest score was 53 out of a maximum possible score of 329; Additional file 1: Table S1), emphasizing that all subjects examined were asymptomatic and none of them met the criteria for FXTAS diagnosis. Nonetheless, the scores were significantly higher in carriers, when compared with controls (23.73 ± 10.40 vs. 12 ± 11.07; *P* = 0.003; Figure 1A), with three outliers – two in the control group and one among the premutation carriers. The boxplot also showed differences in median between both groups (Figure 1B). Taking into consideration that seven tests each at an α level of 5% were performed to assess the group effect on the phenotypes, only the *P* value (0.003) associated with the FXTAS rating score remained significant after Bonferroni correction, showing that the score means between the groups could not be considered equal. In addition, multiple regression analyses taking into account age and CGG repeat numbers explained 52.5% (*R*^2^) of the FXTAS scale score, with *P* <0.0003 and *P* = 0.0014, respectively. Multiple regressions were also run taking into account smoking status, DBP, SBP, and hemoglobin A1c; these variables never appeared significant in any of the analyses.

### Presence of the FXTAS-causative mutation and the number of CGG repeats correlate positively with IL-10 amounts in peripheral blood

**Figure 2:**
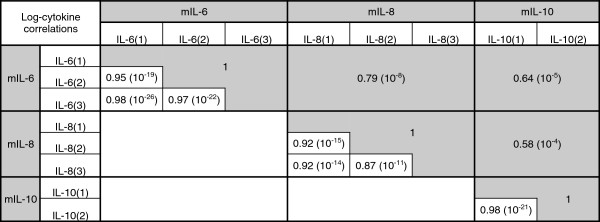
**Correlations between and within the log-transformed cytokine measurements.** Table shows the correlation structure between and within the log-transformed cytokine levels. The Pearson's correlations within a cytokine were computed using repeated measurements. For each of the three cytokines, the mean value was computed based on the repeated measurements and then the correlation between the averaged values was estimated (highlighted in grey). Numbers in brackets reflect the order of magnitude of the *P* values testing the hypothesis of no correlation against the alternative that there is a non-zero correlation.

A set of three cytokines (IL-6, IL-8, and IL-10) was analyzed. This choice was based on previous studies reporting inflammatory changes occurring in somewhat similar neurodegenerative disorders, including AD, PD, and HD [14,26]. The selected cytokines were measured in the supernatants of PBMCs; measurements were carried out in duplicate (for IL-10) or in triplicate (for IL-6 and IL-8). In order to evaluate the quality of the technical replicates, Pearson’s correlations of the repeated measurements for each cytokine were computed. In addition, the correlations between the averaged log-transformed cytokine concentrations were estimated. The correlations between the repeated measurements were all above 0.85, suggesting the use of the average concentrations as a reasonable choice for the modeling analyses (Figure 2).

**Figure 3:**
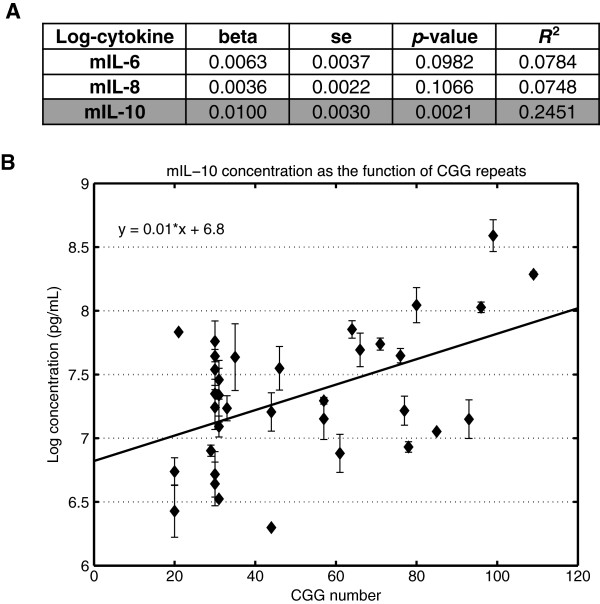
**Effect of CGG repeat length on the mean cytokine concentrations.** (**A**) Each log-transformed averaged cytokine basal concentration was regressed on the number of CGG repeats. Results of the linear modeling include the beta coefficient, the standard error (se), the Student’s *t*-test *P* value associated with the coefficient of the CGG variable and *R*^2^, the fraction of variance explained by the number of CGG repeats. The highlighted results describe the cytokine (mIL-10) for which the number of CGG repeats is significantly associated after multiple testing correction. (**B**) The regression plot represents the logarithm of the mean IL-10 basal concentration (in pg/ml) as the function of the CGG repeats. Each mean value (average of the two repeated measures) is represented by a diamond, and the error bar corresponds to the mean ± standard deviation. The regression line shows the fit of the data for which the equation of regression is also given.

**Figure 4:**
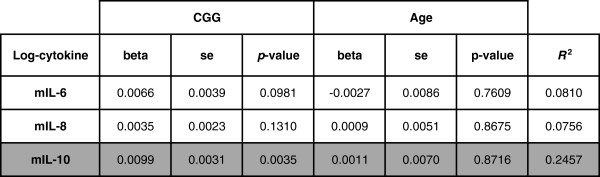
**Effect of the CGG repeats number and age on the mean cytokine concentrations.** Each log-transformed averaged cytokine basal concentration was regressed on the number of CGG repeats and the age variables. Results of the linear modeling include the beta coefficients, the standard errors (se), the Student’s *t*-test *P* values associated with the two coefficients (CGG repeats and age variables) and *R*^2^, the fraction of variance explained by the two variables. The highlighted results describe the cytokine (mIL-10) for which the number of CGG repeats is significantly associated after multiple testing correction.

**Figure 5:**
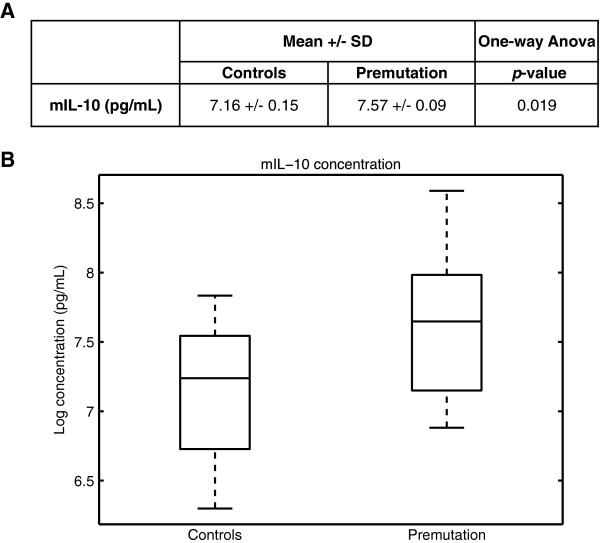
**Group effect on the mean IL-10 concentrations.** (**A**) Mean and standard deviation (SD) of the log-transformed averaged IL-10 concentrations for the control and premutation groups, as well as the *P* value associated with the one-way analysis of variance. (**B**) Boxplot representing the distribution (median and interquartile range) of the mean IL-10 concentrations for control and premutation groups.

We observed a positive linear relationship between the concentration of the anti-inflammatory cytokine IL-10 and the number of CGG repeats (Figure 3A,B). The number of CGG repeats was able to explain 24% (*R*^2^) of the observed variations of the averaged log-transformed IL-10 levels, with *P* = 0.002, which remained significant after Bonferroni multiple testing correction (three tests were considered). Multiple linear regressions modeling of the cytokines’ concentrations as a function of the CGG repeats and age did not indicate any significant effect of age on any of the cytokines studied (Figure 4). In addition, we observed no linear relationship between medication and the levels of cytokines (data not shown). Conversely, the CGG number was significantly correlated with IL-10 concentrations (*P* = 0.0035; Figure 4). We therefore further analyzed the group effect, as shown in Figure 5A,B. A significant increase of IL-10 (*P* = 0.019) was observed in premutation carriers when compared with controls. As all premutation carriers included in this study are asymptomatic, this suggests that the IL-10 variation is an early event occurring prior to classical neurological symptoms.

The relationship between the level of cytokines and FXTAS clinical scores was assessed by regression analysis. No correlation above 0.14 between any of the cytokines and FXTAS scores was observed (data not shown).

## Discussion

We measured the peripheral blood cytokines IL-6, IL-8, and IL-10 in the carriers of the *FMR1* premutation and in control individuals, and found that carriers had significantly higher levels of the anti-inflammatory cytokine IL-10. Concentrations of IL-10 significantly correlated with the number of CGG repeats, whereas the concentrations of proinflammatory cytokines IL-6 and IL-8 did not.

IL-10 is a key orchestrator of the immune system with potent anti-inflammatory effects. An increase in IL-10 concentration in the peripheral blood was previously demonstrated in other neurodegenerative disorders, such as PD and HD [26,27]. Although it is less severe, FXTAS shares features with HD including trinucleotide-repeat expansions, the presence of intranuclear inclusions, abnormal movements and cognitive decline. HD-specific trinucleotide-repeat expansions are thought to primarily cause accumulation of the abnormal polyglutamine-containing proteins [28], unlike FXTAS repeats that affect the 5^′^-UTR of FMR1 mRNA. It is tempting to speculate that an accumulation of the expanded mRNA, which was also reported in HD, plays a role in the activation of specific immune pathways involved in these types of diseases.

In our study, we did not find a significant increase in the concentration of proinflammatory cytokines IL-6 and IL-8 in peripheral blood, as observed in the blood as well as in the brain of patients affected by AD, PD, and HD [29,30]. Bjorkqvist and colleagues compared plasma levels of numerous cytokines in HD patients and observed that the combination of plasma IL-6, IL-8, and IL-10 levels could very efficiently differentiate between HD expansion carriers (premanifest and manifest) and controls [26]. We did not have symptomatic FXTAS patients available for our analysis, and future studies would be necessary to identify if and how the immune status of these individuals changes in the presence of symptoms. However, as shown in Figure 2, we do observe a strong correlation between IL-6 and IL-8 concentrations (*r* = 0.79), whereas the correlation with IL-10 concentrations is weaker (*r* = 0.64 and *r* = 0.58, respectively).

IL-10 is secreted by various cell types: different T-cell subsets, macrophages, dendritic cells, B cells, mast cells, and eosinophils [31-33]. The increased IL-10 levels observed in *FMR1* premutation carriers could therefore be due to activation of immune cells, as well as a direct consequence of IL-10 gene overexpression in PBMCs. Specifically, the *FMR1* gene is expressed in PBMCs [34]; therefore the expression of the pathological repeats could alter intrinsic properties of PBMCs and lead to the increased IL-10 production. In general, cells expressing *FMR1* mRNA containing more than 55 CGG repeats were shown to have growth defects, abnormal distribution of lamin and enhanced expression of stress proteins [4]; these could potentially trigger an increase of IL-10 production in many diverse immune cells. Further studies will be needed to elucidate the precise mechanism of IL-10 production.

Deregulation of IL-10 was associated with various diseases, such as cancer, rheumatoid arthritis, systemic lupus erythematosus, asthma, infectious disorders, and so forth [31], where both overexpression and IL-10 deficiency were shown to have a pathophysiological significance. Most often enhanced expression of IL-10 is beneficial for the individuals due to the activation of anti-inflammatory mechanisms, although a detrimental effect of IL-10 overexpression was demonstrated in a mouse model that subsequently developed a demyelinating peripheral neuropathy [35]. Although FXTAS patients show a reduction of brain myelin content that is also associated with neuronal loss [1], it is hard to say whether this is somehow related to the observed increase of IL-10 levels in peripheral blood.

The relationship between increased concentrations of IL-10 in the peripheral blood of FXTAS patients and central nervous system pathology remains unknown. Pathological changes in the nervous system and increased IL-10 levels in peripheral blood could be independent consequences of CGG-repeat expression in these different compartments. Alternatively, inflammatory activation may originate in the central nervous system, with immunomodulators crossing the brain–blood barrier and reaching the peripheral circulation.

Levels of IL-10 discriminate premutation carriers from controls and may represent a valuable early biomarker. The design of this study including asymptomatic young carriers did not permit any correlation with the progression of the disease. Longitudinal studies including carriers affected with FXTAS are required to address this question.

## Abbreviations

AD: Alzheimer’s disease; CRST: Clinical Rating Scale for Tremor; DSP: diastolic blood pressure; ELISA: enzyme-linked immunosorbent assay; FCS: fetal calf serum; FMR1: fragile X mental retardation 1; FXTAS: fragile X-associated tremor/ataxia syndrome; HD: Huntington’s disease; ICARS: International Cooperative Ataxia Rating Scale; IL: interleukin; PBMC: peripheral blood mononuclear cell; PD: Parkinson’s disease; SBP: systolic blood pressure; UPDRS: Unified Parkinson’s Disease Rating Scale; UTR: untranslated region.

## Competing interests

The authors declare that they have no competing interests.

## Authors' contributions

DM analyzed and contributed to the interpretation of the data, manuscript drafting and revision. SP was involved in the study design, acquired the experimental data, and contributed to data interpretation, manuscript drafting and revision. KE contributed to the acquisition of the experimental data and manuscript revision. JN acquired clinical data, and contributed to data interpretation and manuscript revision. NI contributed to acquisition of the clinical data. AR contributed to the acquisition of the experimental data and manuscript revision. LP contributed to the acquisition of the experimental data. FS contributed to data interpretation, manuscript drafting and revision. GP contributed to data interpretation and manuscript revision. SB contributed to data analyses and interpretation, as well as to manuscript revision. JSB was involved in the study design, and contributed to data interpretation and revision of the manuscript. SJ was involved in the study design, and contributed to acquisition of the clinical data and its interpretation, as well as manuscript revision. GT was involved in the study design, data interpretation, manuscript drafting and revision. All authors read and approved the final version of the manuscript.

## Supplementary Material

Additional file 1 **Table S1.** Dataset used for the study. Presenting, for the 35 subjects involved in this study, the clinical status, age, number of CGG repeats, cytokine measurements (IL-6, IL-8 and IL-10), relevant clinical scores (CRST, UPDRS, ICARS and FXTAS), as well as additional clinical features: diabetes status, smoking status, DBP, SBP, C-reactive protein (CRP) levels, percentage of glycated hemoglobin (HbA1c), treatment status, and the number of psychotropic drugs and pro-tremor/ataxia treatments. Click here for file
